# Nanomaterial-based therapeutic strategies for systemic autoimmune rheumatic diseases: from molecular mechanisms to preclinical translation

**DOI:** 10.3389/fimmu.2026.1981246

**Published:** 2026-10-22

**Authors:** Honghao Guo, Wang Xiang, Liuting Zeng, Wei Xiao, Lingyun Sun

**Affiliations:** 1 Department of Rheumatology and Immunology, Nanjing Drum Tower Hospital, Clinical College of Nanjing Medical University, Nanjing, China; 2 Department of Rheumatology, Changde Hospital, Xiangya School of Medicine, Central South University (The First People's Hospital of Changde City), Changde, China; 3 Department of Rheumatology and Immunology, Nanjing Drum Tower Hospital, Chinese Academy of Medical Sciences & Peking Union Medical College, Nanjing, China

**Keywords:** antifibrotic therapy, clinical translation, immune tolerance, immunomodulation, nanotherapeutics, systemic autoimmune rheumatic diseases

## Abstract

**Background:**

Systemic autoimmune rheumatic diseases (SARDs) are chronic, heterogeneous disorders in which loss of immune tolerance, persistent inflammation, and tissue-specific injury can culminate in irreversible organ damage and fibrosis. Although glucocorticoids, conventional immunosuppressants, biologics, and targeted small molecules have transformed care, long-term treatment remains constrained by systemic toxicity, incomplete tissue selectivity, variable response, and limited ability to restore immune homeostasis.

**Main body:**

Nanomedicine provides a modular framework for improving pharmacokinetics, concentrating therapeutics in diseased tissues, enabling controlled release, and co-delivering immunoregulatory cargos. In this Review, we focus on major SARDs—rheumatoid arthritis (RA), systemic lupus erythematosus (SLE) and lupus nephritis (LN), systemic sclerosis (SSc), Sjögren’s disease (SjD), and idiopathic inflammatory myopathies (IIM)—while using selected evidence from adjacent immune-mediated diseases only when it provides transferable engineering or mechanistic lessons. We critically distinguish passive accumulation from demonstrated target engagement, cargo-mediated effects from intrinsic nanomaterial bioactivity, and reduction of collagen deposition from direct mechanistic evidence of antifibrotic action. We also emphasize that acidic pH, reactive oxygen species (ROS), and matrix metalloproteinase (MMP) activity are not universal SARD features but disease-, organ-, stage-, and patient-dependent variables that require quantitative validation before being used as release triggers. Recent single-cell studies further show that macrophage states form disease- and tissue-specific continua rather than a binary M1/M2 system, requiring more precise cellular endpoints.

**Conclusions:**

Translation of SARD nanotherapeutics will depend less on adding engineering complexity than on demonstrating disease-relevant biodistribution, target engagement, physiologically validated responsiveness, causal mechanism, repeated-dose safety, scalable manufacturing, and regulatory-quality characterization. A disease-adapted, author-proposed framework of complementary evidence domains may support more transparent assessment of preclinical nanoplatforms, without implying a validated linear pathway to clinical use.

## Graphical Abstract

**Figure:**
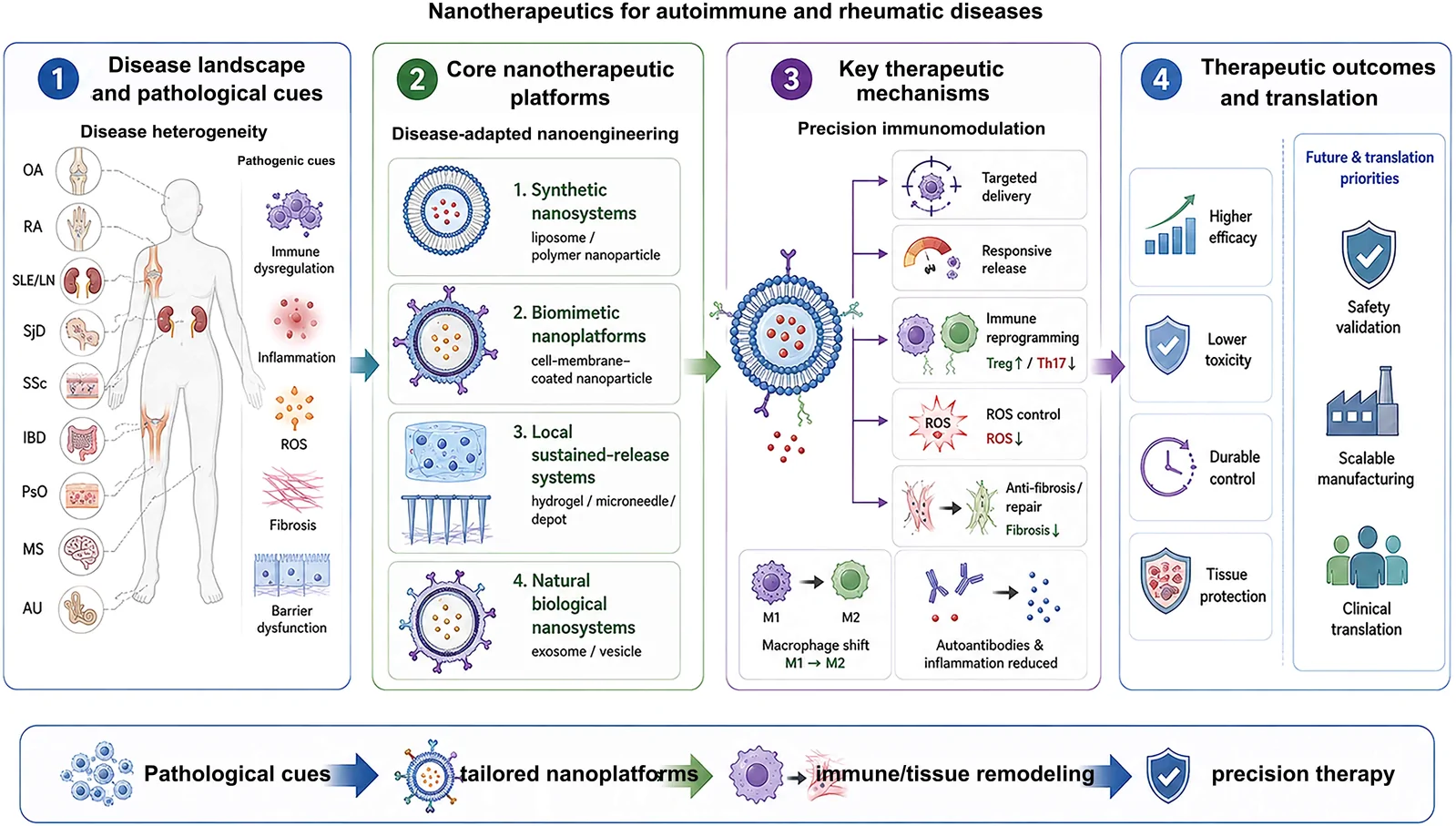

## 1 Background

Systemic autoimmune rheumatic diseases (SARDs) encompass heterogeneous chronic conditions involving dysregulated innate and adaptive immunity, tissue-specific inflammation, and, in some patients, progressive organ injury. The principal diseases considered here are rheumatoid arthritis (RA), systemic lupus erythematosus (SLE), including lupus nephritis (LN), Sjögren’s disease (SjD), systemic sclerosis (SSc), and idiopathic inflammatory myopathies (IIM). Their burden and patterns of organ involvement vary markedly across diseases and individuals (1, 2). Genetic susceptibility and environmental exposures contribute to disease development through context-dependent interactions, although neither category provides a uniform explanation for all SARDs (3, 4). Importantly, established management already includes disease-modifying, treat-to-target, and pathway-directed therapies, as illustrated by contemporary European Alliance of Associations for Rheumatology (EULAR) recommendations for RA and SLE (5, 6). These therapeutic advances provide the appropriate clinical comparator for assessing proposed nanomedicine strategies rather than an assumption that current care is merely symptomatic.

At the mechanistic level, interacting innate and adaptive immune pathways contribute to disease initiation, amplification, and tissue injury, including T follicular helper/regulatory cell imbalance, inflammasome activation, neutrophil extracellular traps, innate lymphoid cells, epigenetic changes, and immunometabolic perturbation (7–12). Their relative contribution varies by clinical phenotype, affected organ, and disease stage. Autoantibody-based clustering further demonstrates biological diversity and overlap across autoimmune rheumatic diseases (13); however, such clustering does not, by itself, establish drug resistance, relapse rates, or determinants of treatment response. Heterogeneous responses to treatment should instead be understood in the context of disease-specific therapeutic evidence, patient characteristics, prior exposure, comorbidity, and the treatment target (5, 6).

Despite substantial progress in therapeutic development, important unmet needs persist for particular SARD populations, including refractory organ manifestations, residual inflammatory activity, cumulative treatment-related toxicity, and the difficulty of achieving sustained treatment-free remission. These problems differ by drug, disease, and clinical setting and should not be attributed uniformly to every existing treatment (5, 6). Reviews of nanotechnology-enabled therapies have identified potential formulation and delivery opportunities (14, 15), but these do not negate the established efficacy of conventional or targeted disease-modifying agents. Glucocorticoids illustrate a treatment-specific trade-off: they can control acute inflammation effectively, while prolonged exposure may produce dose- and duration-related adverse events, supporting steroid-sparing strategies where clinically feasible (6, 16). Conventional synthetic disease-modifying antirheumatic drugs (csDMARDs), such as methotrexate and hydroxychloroquine, are pharmacologically heterogeneous; poor aqueous solubility, instability, restricted bioavailability, and nonspecific tissue distribution are relevant to selected compounds or formulations, not shared defining features of the entire class. Proposed nanocarriers should therefore be evaluated against the pharmacology and clinical performance of the particular agent they aim to improve, rather than against a generalized model of csDMARD failure.

Biologic therapies and targeted synthetic agents have expanded pathway-directed treatment and improved clinical outcomes in selected SARDs (5, 6). Monoclonal antibodies, fusion proteins, and small-molecule inhibitors differ in target breadth, administration schedule, half-life, immunogenic potential, tissue penetration, and safety profile. Some biologics can elicit anti-drug antibodies or infusion/injection reactions, while infection risk and other adverse effects depend on the specific mechanism and patient context; it would be inaccurate to portray all biologics as short-lived, poorly distributed, or intrinsically immunogenic to the same degree. Nor is the fact that an agent has a defined molecular target sufficient to establish clinical inadequacy, because pathway-selective therapies can provide substantial disease modification. Current strategies already aim for immunological remission and organ protection, and emerging tolerance-directed approaches seek more durable immune reprogramming (17). The unresolved challenge is to extend effective, safe, and individualized disease control to patients and manifestations with unmet needs—not to claim that existing care provides only passive symptom relief or that eradication of every SARD is a realistic near-term benchmark.

The rapid evolution of nanobiomedicine offers a technological route to address several pharmacokinetic and tissue-delivery limitations of conventional SARD therapy (18, 19). Nanocarriers can improve aqueous solubility, protect labile cargos, extend circulation, and enable controlled or co-delivery of multiple therapeutics (20, 21). However, claims of ‘targeting’ require careful interpretation. Increased signal within inflamed tissue can result from prolonged circulation, vascular leak, phagocyte sequestration, or true ligand–receptor recognition, and therefore accumulation alone should not be equated with cellular target engagement. Active targeting should ideally be supported by quantitative biodistribution, receptor-dependence or competition experiments, cell-subset uptake, and evidence that delivery changes the intended molecular pathway at the disease site. This distinction is particularly important in SARDs, where inflamed tissues, reticuloendothelial organs, and normal immune compartments can all take up nanomaterials (20, 21).

Recent preclinical studies have nevertheless demonstrated that rational nanoplatform design can improve local drug exposure and modulate pathogenic immune circuits in selected SARD models. Lipid, polymeric, dendrimer, biomimetic, and extracellular-vesicle platforms have been evaluated most extensively in RA and SLE/LN, with emerging studies in SSc and more limited disease-specific evidence in SjD and IIM. The biological contribution of the carrier itself must also be separated from that of the payload: some dendrimers, ceria-based materials, extracellular vesicles, and cell-membrane coatings possess intrinsic anti-inflammatory, antioxidant, or immunoregulatory activities. Appropriate controls therefore need to include unloaded carrier, free cargo, non-targeted carrier, and, where relevant, trigger-insensitive formulations to determine which component accounts for therapeutic benefit.

Despite rapid growth, the SARD nanotherapeutics field remains limited by fragmented mechanistic evidence and a substantial gap between proof-of-concept efficacy and clinically informative validation. Many studies use a single induced rodent model, short treatment windows, and surrogate inflammatory endpoints. Long-term pharmacokinetics, repeated-dose tolerability, immunogenicity, degradation products, organ accumulation, and effects on protective immune function remain incompletely characterized. Moreover, disease-associated triggers used for responsive release—including acidic pH, reactive oxygen species (ROS), and protease activity—are neither universal nor uniform across SARDs. Their magnitude varies by disease, organ, disease stage, vascularity, concomitant treatment, and patient. These limitations require a more critical framework that distinguishes engineering feasibility from disease-specific therapeutic evidence.

Accordingly, this Review focuses on RA, SLE/LN, SSc, SjD, and IIM. Evidence from osteoarthritis, gout, inflammatory bowel disease, psoriasis, multiple sclerosis, or autoimmune uveitis is not treated as direct SARD efficacy evidence; when retained, it is identified as transferable engineering or mechanistic information requiring disease-specific revalidation. We examine which delivery problems different nanoplatforms might address, how distribution and target engagement are documented, whether observed efficacy is causally attributable to cargo and/or carrier, and which safety and quality assessments are relevant to the intended product. The comparisons are intended to inform study design, not to rank all platform classes by therapeutic superiority.

This disease-centered framework links nanomaterial properties to SARD-specific pathology and to the evidentiary standards required for translation (**Figure 1**).

**Figure 1:**
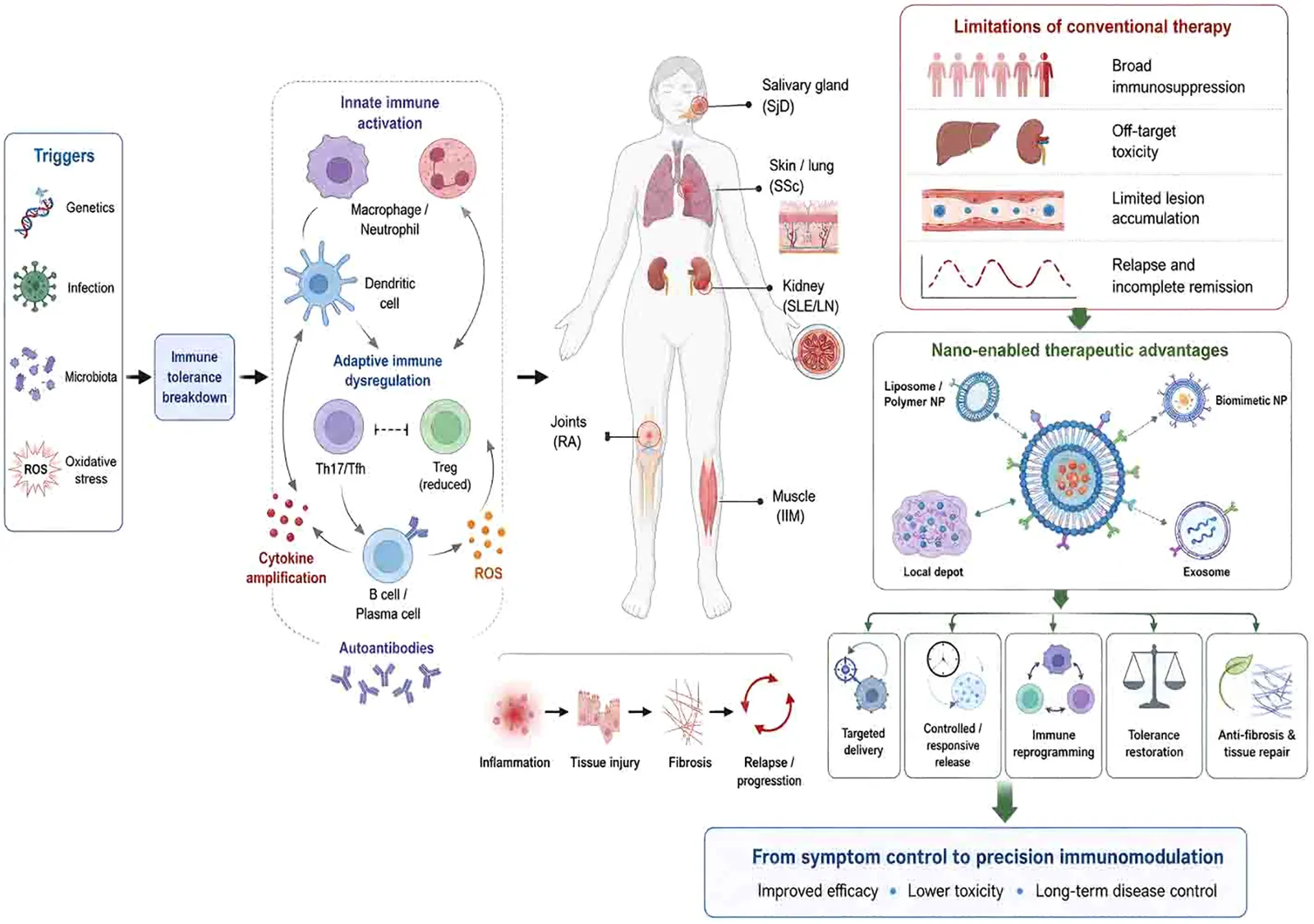
Pathological landscape of systemic autoimmune rheumatic diseases and the rationale for nanotherapeutics. Genetic susceptibility, infection, microbiota dysbiosis, and oxidative stress can disrupt immune tolerance and promote coordinated dysregulation of innate and adaptive immunity. Aberrant activation of macrophages, neutrophils, and dendritic cells, together with imbalances among Th17/Tfh cells, Tregs, and B cells/plasma cells, drives cytokine amplification, autoantibody production, and excessive ROS generation. These interconnected immune abnormalities contribute to persistent inflammation, tissue injury, fibrosis, and recurrent or progressive organ damage involving the joints, kidneys, salivary glands, skin, lungs, and muscles. Although established disease-modifying and targeted therapies can achieve substantial clinical benefit, selected agents and patient groups face limitations related to systemic adverse effects, incomplete response, or insufficient exposure in particular affected tissues. Nanotherapeutic platforms, including liposomal and polymeric nanoparticles, biomimetic nanoparticles, local nanodepots, and extracellular-vesicle-based systems, are being investigated for cargo protection, localized or controlled delivery, and modulation of selected immune or fibrotic pathways, with efficacy and specificity remaining formulation- and model-dependent. The schematic summarizes proposed opportunities that require direct validation against established disease-modifying treatments; it does not imply that current care consists only of symptomatic management. IIM, idiopathic inflammatory myopathy; LN, lupus nephritis; NP, nanoparticle; RA, rheumatoid arthritis; ROS, reactive oxygen species; SARDs, systemic autoimmune rheumatic diseases; SjD, Sjögren’s disease; SLE, systemic lupus erythematosus; SSc, systemic sclerosis; Tfh, T follicular helper cell; Th17, T helper 17 cell; Treg, regulatory T cell.

Our goal is therefore not to portray nanotechnology as intrinsically superior to established therapy, but to identify where nano-enabled pharmacology provides a testable advantage, where current claims exceed the evidence, and which experimental and regulatory steps are needed to move the field toward safer, mechanism-based precision treatment.

## 2 Classification and design of core nanotherapeutic platforms for SARDs

SARD treatment now encompasses multiple effective drug classes, and potential delivery limitations should be judged for individual agents, doses, organs, and clinical objectives (5, 6). The value of nano-enabled formulations may lie in solving a demonstrable problem—such as inadequate exposure of a labile cargo, dose-limiting systemic exposure, a need for a localized depot, or combined delivery of mechanistically complementary agents—rather than assuming that conventional therapy is uniformly nonspecific or inferior. Because the relative benefits and liabilities are formulation-dependent, comparisons should be matched to the active ingredient, route, disease context, and relevant standard-of-care formulation. Where causal attribution is important, comparator arms can include the free drug, unloaded carrier, and appropriate targeting or responsiveness controls.

SARD nanotherapeutic design must also account for marked heterogeneity in the pathological microenvironment. Acidification, oxidative stress, protease activity, vascular permeability, chemokine gradients, extracellular-matrix remodeling, and fibrosis can occur in inflamed tissues, but their magnitude and spatial distribution differ substantially among RA synovium, lupus kidney, scleroderma skin or lung, SjD salivary glands, and inflamed muscle. Even within one disease, these cues change with activity, chronicity, organ compartment, and treatment. Accordingly, pH-, ROS-, enzyme-, or matrix-responsive systems should be described as disease-context-dependent engineering strategies rather than universal SARD platforms, and their trigger thresholds should be validated against measured physiological and pathological ranges.

Based on material source, structural biomimicry, release architecture, and biological interface, we group the principal platforms into four categories: synthetic functional nanosystems, cell-membrane-biomimetic nanoplatforms, localized sustained-release systems, and natural biological nanotherapeutics. This classification is intended as an analytical framework rather than a hierarchy of superiority. Each class solves different delivery problems while introducing distinct limitations in biodegradation, immune recognition, manufacturing, quality control, repeated-dose safety, and regulatory development. The clinically relevant question is therefore not which platform is ‘best’, but whether a given design provides a reproducible advantage for a defined disease, organ, cell state, and therapeutic cargo. The overall classification and design logic of these platform classes are summarized in **Figure 2**.

**Figure 2:**
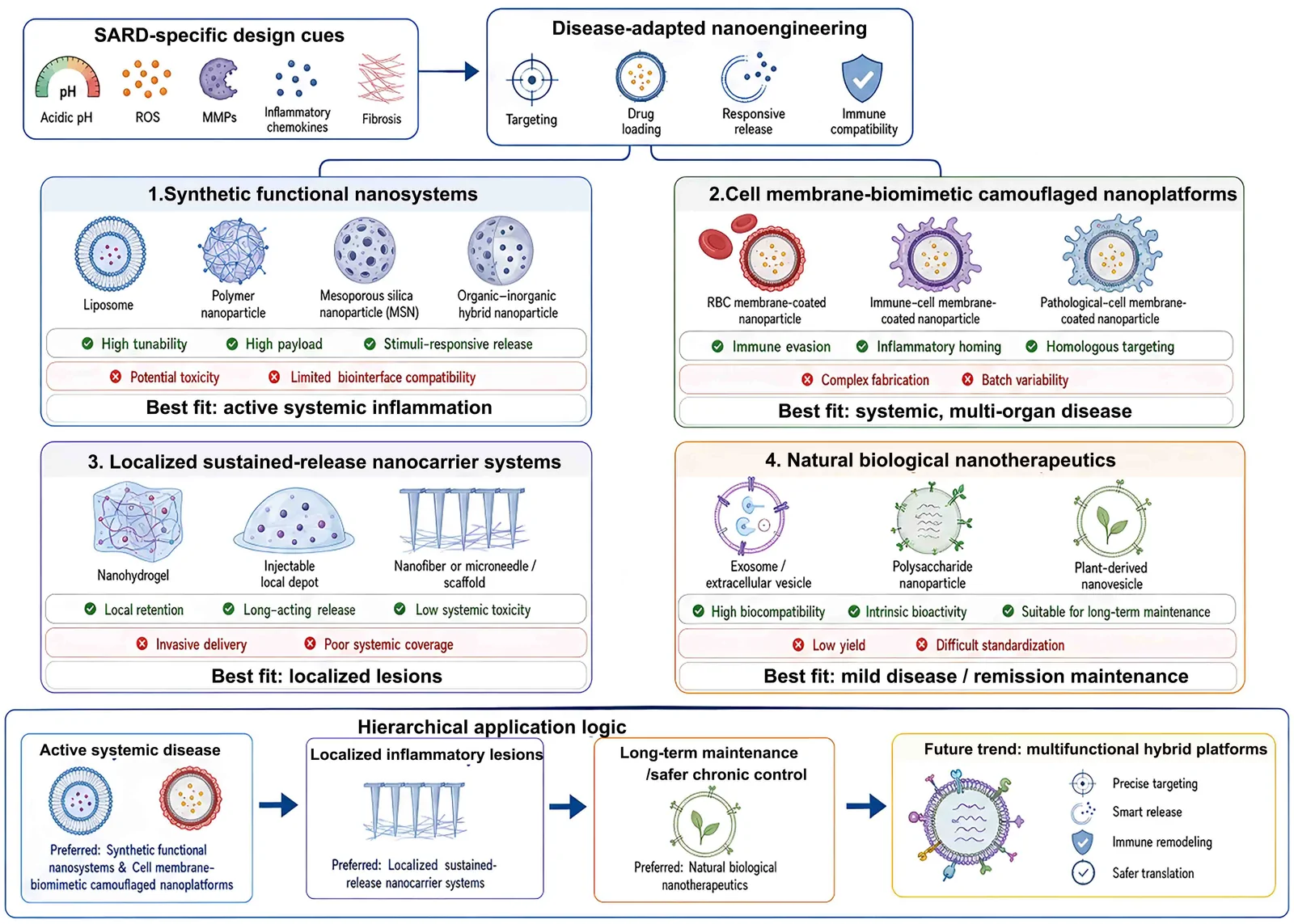
Classification and design logic of major nanotherapeutic platforms for systemic autoimmune rheumatic diseases. Four broad platform classes are compared: synthetic functional nanosystems, cell-membrane-biomimetic nanoplatforms, localized sustained-release systems, and natural biological nanotherapeutics. Disease-associated features such as pH, reactive oxygen species (ROS), matrix metalloproteinase (MMP) activity, vascular permeability, chemokines, and fibrosis can inform formulation design, but these cues are not universal SARD characteristics and vary by disease, organ, stage, treatment, and patient. Synthetic systems offer modular control of composition and release but may introduce material-specific toxicology and manufacturing complexity. Biomimetic systems can modify circulation and biological interactions but require validation of membrane identity, orientation, target engagement, and batch consistency. Local depots can increase exposure within accessible lesions but provide limited coverage of systemic disease and introduce local retention and procedure-related risks. Natural biological systems may provide endogenous bioactivity but are compositionally complex and difficult to standardize. Platform selection should therefore be driven by the therapeutic problem, route, target compartment, cargo, chronicity, safety, and manufacturability rather than by a presumed hierarchy of efficacy or biocompatibility. MMPs, matrix metalloproteinases; MSN, mesoporous silica nanoparticle; RBC, red blood cell; ROS, reactive oxygen species; SARDs, systemic autoimmune rheumatic diseases.

### 2.1 Synthetic functional nanosystems

Synthetic functional nanosystems include inorganic, organic, and hybrid nanocarriers constructed through chemical synthesis, self-assembly, or surface functionalization. Their principal strength is engineering control over particle size, morphology, charge, surface chemistry, drug loading, ligand density, and release behavior (22). This modularity enables pharmacokinetic optimization and co-delivery of small molecules, proteins, or nucleic acids, but it also creates formulation-specific liabilities. Surface chemistry can alter protein-corona formation and immune recognition; some inorganic components may persist in reticuloendothelial organs; and increasingly complex multi-component systems can become difficult to manufacture reproducibly. Thus, material tunability should be considered an enabling property rather than evidence of biological superiority.

#### 2.1.1 Organic synthetic nanosystems

Organic synthetic nanosystems include liposomes, polymeric nanoparticles and micelles, dendrimers, and nanohydrogels. These carriers can accommodate chemically diverse cargos and can be modified with polyethylene glycol, targeting ligands, or responsive linkers (23, 24). Prolonged circulation or inflammatory-site accumulation is sometimes attributed to an enhanced permeability and retention (EPR)-like effect; however, vascular leak and phagocytic uptake are highly variable in inflammatory disease and should not be equated with active targeting. For SARDs, organ-level fluorescence therefore needs to be complemented by quantitative biodistribution, cell-subset uptake, and—where a ligand or receptor is invoked—evidence of receptor-dependent engagement.

Polymeric nanoparticles and micelles can use biodegradable matrices such as poly(lactic-co-glycolic acid) (PLGA) or hyaluronic-acid-derived materials, whereas polyethylene glycol (PEG) is commonly employed as a hydrophilic surface modifier rather than a degradable matrix. PEGylation can reduce some forms of protein adsorption and alter circulation time, although its performance and immunological consequences depend on formulation and prior host exposure. Dendrimers offer dense, chemically addressable surfaces for nucleic-acid complexation and ligand display. In the fluorinated polyamidoamine dendrimer/miR-23b study in experimental RA, treatment reduced TAB2, TAB3, and IKKα at transcript and protein levels together with downstream inflammatory mediators (25). We therefore interpret the findings as pathway-consistent attenuation of nuclear factor kappa B (NF-κB) signaling through validated upstream targets, rather than as proof that every step of NF-κB activation was directly interrogated. The carrier itself may also contribute biological activity, making unloaded-carrier controls essential for mechanistic attribution.

#### 2.1.2 Inorganic synthetic nanosystems

Inorganic synthetic nanosystems include mesoporous silica nanoparticles (MSNs), metal and metal-oxide nanoparticles, carbon-based materials, and other nanostructures. Their physicochemical properties can support high surface loading, catalytic activity, imaging, or externally triggered behavior. These features can be useful when they solve a defined therapeutic problem, but they also raise material-specific questions regarding dissolution, persistence, protein adsorption, oxidative effects, and long-term organ retention. Consequently, claims of superior stability or multifunctionality should be balanced against biodegradation and repeated-dose toxicology, particularly in chronic diseases that may require years of treatment.

Metal-oxide systems illustrate why carrier and cargo effects must be separated. Cerium oxide nanoparticles can display redox-enzyme-mimetic activity and thereby alter oxidative stress independently of any loaded drug. In a 2025 RA study, silver-modified ceria nanoparticles carrying celastrol were reported to reduce joint inflammation while combining ROS scavenging with celastrol delivery (26). Such multi-active constructs are attractive, but efficacy cannot automatically be assigned to improved delivery: unloaded nanozyme, free-drug, loaded-carrier, and composition-matched controls are needed to determine the relative contributions of intrinsic nanomaterial activity and cargo pharmacology.

#### 2.1.3 Organic-inorganic hybrid nanosystems

Organic–inorganic hybrid nanosystems combine an engineered organic interface with an inorganic core or catalytic component. This architecture can integrate cargo loading, surface functionalization, redox activity, imaging, or stimulus responsiveness within one formulation (27). The same multifunctionality, however, complicates causal interpretation and manufacturing. The 2025 celastrol-loaded silver-modified ceria platform in RA exemplifies this trade-off: the inorganic component has intrinsic redox activity, whereas celastrol provides independent anti-inflammatory pharmacology (26). For hybrid systems, mechanistic deconvolution and a simplified composition that preserves only components required for efficacy may be more important for translation than maximizing the number of functions.

### 2.2 Cell membrane-biomimetic camouflaged nanoplatforms

Cell-membrane-biomimetic nanoplatforms use natural membrane components to modify the biological interface of a synthetic or drug-containing core. Their rationale is to retain selected source-cell proteins or lipids that may reduce nonspecific clearance or promote interactions with inflamed endothelium and immune cells (28, 29). These systems are promising but should not be assumed to provide immune invisibility or disease-specific homing. Membrane isolation, orientation, protein retention, source-cell activation state, and batch variability can all alter function, and residual biological material can itself be immunologically active.

The principal advantage of membrane biomimicry is therefore not exact reproduction of a source cell, but partial retention of selected biological interface functions. Adhesion molecules, chemokine receptors, or ‘self’ signals may influence circulation and tissue interaction, yet coating efficiency and functional orientation require direct characterization. Apparent lesion enrichment can arise from prolonged circulation, vascular leak, or mononuclear-phagocyte uptake rather than receptor-specific targeting. Studies claiming active homing should therefore incorporate quantitative biodistribution and, when feasible, receptor-blocking, competition, or loss-of-function controls.

#### 2.2.1 Red blood cell membrane-camouflaged nanoplatforms

Red blood cell (RBC) membrane-camouflaged nanoparticles are a frequently studied biomimetic approach because RBC membranes contain endogenous ‘self’ signals, including CD47, that can modulate phagocytic recognition. Their principal rationale is to alter the nano–bio interface and potentially prolong circulation; neither prolonged circulation nor disease-site targeting should be assumed from the membrane source alone. Membrane integrity, protein orientation, core composition, dose, and host immune status can all modify disposition. Quantitative pharmacokinetic and biodistribution studies are therefore required for each formulation.

RBC membrane coating is primarily a circulation-extending and immune-evasive strategy rather than an inherently lesion-specific targeting mechanism. Quantitative effects should therefore be reported at the level of the individual formulation and disease model instead of generalized across SARDs. In an MRL/lpr mouse model of SLE, RBC membrane-coated PLGA nanoparticles carrying cyclosporine A showed a 2.63-fold longer plasma half-life and a 4.87-fold higher area under the concentration–time curve (AUC) than free cyclosporine A, together with improved therapeutic activity (30). These pharmacokinetic gains support the value of membrane camouflage, but they do not establish a universal three- to fivefold increase in lesion accumulation or a fixed reduction in systemic adverse effects across RA and SLE. More convincing evidence of targeting would require quantitative tissue and cell-subset biodistribution, comparison with uncoated controls, and demonstration of engagement with the intended pathological compartment.

#### 2.2.2 Immune cell membrane-camouflaged nanoplatforms

Immune-cell-membrane platforms derived from monocytes, macrophages, neutrophils, or other leukocytes are designed to exploit selected adhesion or chemotactic features of inflammatory cells. Recent RA studies provide proof of principle that membrane-coated nanocrystals can increase joint localization and therapeutic activity, but the mechanism of enrichment can combine prolonged circulation, inflammatory vascular access, and membrane-mediated interactions rather than a single ‘active-targeting’ process (29, 31). Accordingly, claims of superior targeting should be supported by quantitative comparisons with uncoated or composition-matched controls and, where a receptor mechanism is proposed, by blocking or competition experiments.

Immune-cell-membrane platforms may additionally carry receptors or adhesion molecules capable of binding inflammatory mediators or activated endothelium. However, descriptions of macrophage ‘M1-to-M2 polarization’ should be interpreted cautiously. The M1/M2 terminology is useful as a simplified *in vitro* shorthand, but *in vivo* rheumatic tissues contain overlapping inflammatory, tissue-resident, remodeling, interferon-responsive, and resolution-associated macrophage states rather than two discrete populations. Accordingly, nanotherapy studies should define macrophage responses using multi-marker, transcriptional, spatial, and functional readouts rather than relying on a small number of M1/M2 markers (29).

However, these sophisticated biomimetic systems have important technical and translational limitations. Isolation of immune cells and purification of their membranes are complex and difficult to scale; membrane activity is sensitive to manufacturing conditions, resulting in batch variability; activated immune-cell membranes may retain pro-inflammatory signals and potentially trigger mild inflammatory activation; and the *in vivo* metabolism and long-term immune safety of immune-cell membrane coatings remain incompletely defined. These limitations substantially constrain clinical translation (28).

#### 2.2.3 Pathological cell membrane-camouflaged nanoplatforms and critical perspectives

Membranes derived from disease-relevant cells have also been proposed as homotypic interfaces, on the premise that retained adhesion molecules may enhance interaction with related pathological cells. This concept is mechanistically interesting but remains substantially less validated in SARDs than RBC- or leukocyte-membrane approaches. Evidence from bio-inspired delivery systems should therefore be treated as an engineering hypothesis until disease-specific receptor dependence, quantitative target-cell engagement, off-target distribution, and batch reproducibility are demonstrated (32).

Cell-membrane biomimicry also introduces translational liabilities that become more important during chronic treatment. Long-term accumulation, membrane-protein metabolism, repeated-dose immunogenicity, complement activation, and effects of residual biological material remain insufficiently characterized (21). Manufacturing adds further complexity because source-cell state, isolation, membrane purity, coating efficiency, orientation, and storage can alter the biological interface. Rather than increasing complexity through multiple membrane sources by default, future development should prioritize functionally justified components, quantitative identity and potency assays, scalable processing, and direct demonstration that the membrane contributes a reproducible advantage over a simpler carrier (32, 33).

### 2.3 Localized sustained-release nanocarrier systems

Localized sustained-release nanocarriers aim to increase residence time and drug exposure within accessible lesions while reducing systemic exposure. The approach is most developed for intra-articular therapy in RA, where hydrogels, particulate depots, or *in situ* gelling systems can retain therapeutics within the joint and support controlled release (34). This strategy is fundamentally different from systemic targeting: it trades whole-body coverage for local pharmacokinetic control. Its value therefore depends on whether the clinically important disease burden is sufficiently localized and whether repeated local administration is acceptable.

#### 2.3.1 Structural design and sustained-release mechanisms

Localized systems can use nanohydrogels, porous scaffolds, nanofibers, microneedles, or injectable depots to regulate release by diffusion, degradation, reversible binding, or stimulus-sensitive crosslinks (34). The relevant design metrics are not merely prolonged release in buffer but retention in the target tissue, exposure of the active cargo, mechanical compatibility, local inflammatory response, and reproducibility under disease-relevant fluid turnover. Responsive local systems should also be challenged with trigger-insensitive controls and with physiological concentrations of the proposed stimulus.

Localized systems can additionally incorporate pH-, ROS-, enzyme-, or other microenvironment-responsive chemistries to couple drug release to local inflammatory conditions. However, proof of responsiveness in an artificial buffer is insufficient to establish disease specificity. Trigger thresholds, release kinetics, and premature leakage should be tested in protein-containing media and disease-relevant biofluids across overlapping normal and pathological ranges, together with a trigger-insensitive control (34). Because inflammatory cues change over time and vary among patients, responsive release should be described as a calibrated pharmacological feature rather than an automatic readout of disease activity.

#### 2.3.2 Advantages and disease-specific adaptability for SARDs

Local delivery may reduce systemic drug exposure, but lower systemic exposure should not be equated with established long-term safety. Intra-articular or implanted materials can undergo burst release, washout, foreign-body reactions, local fibrosis, infection, or degradation into active products; repeated procedures can add cumulative risk. Comparative studies should therefore report local and systemic pharmacokinetics, joint or tissue residence, structural toxicity, immune responses to the material, and functional outcomes rather than relying only on reduced serum drug concentration (34).

The strongest current rationale for localized nanotherapy is in joint-dominant RA, where intra-articular depots can directly address synovial inflammation and drug residence (34). Related local concepts may be explored for cutaneous SSc or glandular SjD, but disease-specific evidence is less mature and should not be inferred from RA. Moreover, local therapy cannot substitute for systemic disease control when clinically important pathology is multisystem or spatially inaccessible. Route selection should therefore follow organ distribution and treatment goals rather than material convenience.

#### 2.3.3 Bottlenecks in clinical translation

From a translational perspective, local sustained-release systems face several constraints: physiological fluid turnover can shorten residence time; implanted or slowly degradable materials can provoke foreign-body responses; degradation products may alter local inflammation; and repeated injection or implantation can cause tissue injury or infection. These risks are especially relevant for chronic SARD treatment and should be assessed together with local biomechanics, tissue function, systemic leakage, and repeated-dose tolerability (21, 34). Future optimization should focus on reproducible retention, complete or predictable clearance, minimally invasive administration, and evidence that the longer local exposure produces clinically meaningful benefit relative to simpler formulations.

### 2.4 Natural biological nanotherapeutics

Natural biological nanotherapeutics include extracellular vesicles (EVs), cell-derived vesicles, plant-derived nanovesicles, and other bio-derived nanoscale systems. Their biological origin can provide complex membrane and cargo functions that are difficult to recreate synthetically, and bio-inspired delivery systems may offer useful interfaces for immune modulation or tissue repair (32, 33). However, ‘natural’ should not be treated as synonymous with safe, non-immunogenic, or clinically standardized. Source variability, donor or culture conditions, isolation methods, co-isolated contaminants, endogenous bioactive cargos, storage instability, and poorly defined dose metrics can substantially alter pharmacology and safety.

#### 2.4.1 Exosome-based natural nanotherapeutics

EVs are lipid-bilayer particles secreted by cells that transport proteins, lipids, and nucleic acids and can modify recipient-cell behavior. Mesenchymal stromal cell-derived EVs have been investigated in autoimmune and inflammatory models because they can carry immunoregulatory and reparative signals, while engineered EVs can be modified to enrich therapeutic cargo or improve tissue interaction (33). Their biological complexity is also a challenge: potency can vary with cell source and manufacturing conditions, and observed efficacy may reflect endogenous vesicle cargo, an engineered payload, altered biodistribution, or a combination of these mechanisms.

EV-based therapy may avoid some risks associated with administering viable cells, but it introduces a different set of translational uncertainties. These include heterogeneity of vesicle subpopulations, incompletely defined active components, residual proteins or nucleic acids, scalable purification, batch-to-batch potency, storage, biodistribution, and immunological effects during repeated dosing (33). Consequently, long-term maintenance use should not be assumed to be safe on the basis of biological origin alone. Engineered EV studies should use unmodified-vesicle, free-cargo, and appropriately matched process controls whenever possible.

#### 2.4.2 Natural polysaccharide and phytogenic nanosystems

Plant-derived vesicles and polysaccharide-based nanomaterials expand the range of bio-derived systems under investigation. Some polysaccharides can provide biodegradable matrices for macromolecular delivery, while plant-derived vesicles may carry lipids, proteins, and nucleic acids with biological activity (23, 33). Their composition is strongly source- and process-dependent, and pharmacologically active endogenous constituents can confound attribution of carrier versus cargo effects. Standardized source materials, compositional profiling, potency assays, and contaminant control are therefore prerequisites for meaningful cross-study comparison.

Potential advantages of bio-derived nanosystems include renewable source materials and compatibility with low-temperature or aqueous processing, but claims of low cost, high safety, or suitability for chronic use require product-specific evidence. Manufacturing yield, purification burden, storage, biological variability, and regulatory classification may offset apparent advantages at laboratory scale (32, 33). Hybridization with synthetic polymers, ligands, or nucleic-acid cargos can increase functionality but also increases the number of critical quality attributes that must be controlled.

#### 2.4.3 Critical challenges and translational prospects of natural nanotherapeutics

Natural biological nanotherapeutics remain attractive because they can combine delivery with endogenous biological functions, but the field is still constrained by incomplete product definition. Multiple active components can make mechanism and dose difficult to standardize; biological source, culture or growth conditions, isolation methods, and storage can alter composition and potency; and absorption, distribution, metabolism, excretion, and repeated-dose immunological effects remain incompletely characterized (21, 32, 33). Translation therefore requires the same rigor applied to synthetic nanomedicines: defined identity, quantitative potency, contaminant control, scalable manufacture, pharmacokinetics, and chronic safety rather than an assumption of compatibility based on biological origin.

Future development of natural biological nanotherapeutics should prioritize standardized and scalable manufacture, quantitative identity and potency assays, definition of active components, reproducible engineering of targeting or cargo, and repeated-dose pharmacology and safety. Progress will depend on demonstrating a specific advantage over simpler formulations rather than presuming superior compatibility from biological origin.

### 2.5 Comparative platform selection according to delivery requirements

No nanoplatform class is intrinsically suited to a particular disease severity, treatment phase, or SARD diagnosis. Platform selection should begin with a defined clinical or pharmacological delivery problem: the intended organ and cellular compartment, the properties and stability of the therapeutic cargo, the preferred administration route, required exposure duration, the feasibility of local versus systemic treatment, and the risks associated with repeated use. Comparisons should be made against an appropriate formulation of the same active agent or another clinically relevant comparator, rather than against a hypothetical uniformly inadequate conventional therapy.

Synthetic lipid, polymeric, dendrimer, and hybrid carriers can protect labile cargo, tune release, or enable multidrug delivery, but their composition and surface chemistry affect protein adsorption, clearance, degradation, and manufacturing reproducibility. Cell-membrane-coated carriers may alter biological interactions yet require source characterization, retained membrane-function assays, and controls that distinguish coating effects from simple prolonged circulation. Local depots address residence at accessible lesions but are not substitutes for systemic management when multiple organs are affected. Extracellular vesicles and other biologically derived systems may contribute active endogenous components, which complicate potency measurement and separation of carrier from cargo effects.

Accordingly, a defensible choice balances mechanistically demonstrated exposure or functional benefit against material persistence, host immune interactions, route-specific risks, production scale, and product-specific quality attributes. Where direct head-to-head data are absent, platform comparisons remain qualitative engineering assessments, not evidence-based clinical rankings. **Table 1** summarizes the principal design trade-offs and delivery problems potentially addressed by the four platform categories.

**Table 1:** Comparative nanoplatform attributes and delivery problems potentially addressed (not evidence-based disease-severity assignments).

Platform category	Representative systems	Core design features	Major advantages	Key limitations	Relevant delivery problem (not disease severity)
Synthetic functional nanosystems	Liposomes; polymeric nanoparticles and micelles; dendrimers; mesoporous silica nanoparticles; metal/metal oxide nanoparticles; organic-inorganic hybrids	Tunable size, charge and surface chemistry; high drug loading; ligand functionalization; pH/ROS/enzyme-responsive release; single- or multi-cargo delivery	High structural controllability; modular engineering; improved cargo solubility/stability; potential pharmacokinetic optimization and combination delivery	Possible cytotoxicity or hemotoxicity; immune recognition; incomplete biodegradation of some inorganic systems; uncertain long-term accumulation; complex hybrid manufacturing	Formulation of unstable/poorly soluble cargos; tunable release; local or systemic exposure; combination delivery when justified by cargo properties and route.
Cell membrane-biomimetic nanoplatforms	Red blood cell-, macrophage-, neutrophil-, T-cell-, platelet- or pathological-cell membrane-coated nanoparticles; hybrid membrane systems	Natural membrane shell plus functional nano-core; preservation of adhesion molecules, chemokine receptors and immune-evasion signals; homologous or inflammation-homing targeting	Potential to modify circulation and biological interactions; may support inflammatory-tissue or cell engagement when source-membrane functions are retained and validated	Complex membrane isolation and coating; batch variability; possible residual inflammatory signals; membrane instability; unclear long-term metabolism and immune safety	Biological-interface engineering to investigate altered circulation, adhesion, or cellular uptake; requires source-consistency and mechanism-specific verification.
Localized sustained-release nanocarriers	Nanohydrogels; injectable depots; porous scaffolds; nanofibers; *in situ* gelling systems; microneedle-based local platforms	Local implantation/injection/topical delivery; prolonged retention; diffusion- or degradation-controlled release; optional microenvironment responsiveness	High local exposure with lower systemic exposure; prolonged local residence; potential reduction in off-target exposure for accessible focal lesions	Limited value for diffuse systemic disease; local washout or retention failure; invasive administration for some systems; foreign-body reactions or local fibrosis; infection risk with repeated procedures	Sustained exposure at accessible focal sites; localized dosing and retention where systemic disease coverage is not the sole therapeutic objective.
Natural biological nanotherapeutics	Exosomes and other extracellular vesicles; MSC-derived vesicles; natural polysaccharide nanoparticles; plant-derived nanovesicles; other bio-derived nanosystems	Biological cargo and membrane composition confer intrinsic immunomodulatory or tissue-repair activity; can be further engineered for targeting or cargo enrichment	Potential biological compatibility and biodegradability; endogenous immunoregulatory or reparative cargo in some systems; amenable to bioengineering	Complex composition; variable potency; difficult quality control and standardization; limited production yield for some vesicle systems; storage instability; incompletely defined pharmacokinetics and long-term effects	Delivery or intrinsic bioactivity requiring defined identity, potency and source-controlled manufacture; proposed applications remain product-specific.

MSC, mesenchymal stromal cell; ROS, reactive oxygen species.

## 3 Preclinical evidence in core systemic autoimmune rheumatic diseases

The preclinical literature is highly uneven across SARDs. RA and SLE/LN account for most mechanistically developed nanomedicine studies, whereas SSc has a smaller but rapidly expanding antifibrotic literature and disease-specific nanotherapy in SjD and IIM remains comparatively sparse. We therefore separate direct SARD evidence from evidence borrowed from adjacent inflammatory diseases and avoid treating all immune-mediated conditions as interchangeable. A multilevel overview of the principal therapeutic mechanisms is summarized in **Figure 3**, whereas disease-adapted strategies across core SARDs and transferable immune-mediated contexts are organized in **Figure 4**.

**Figure 3:**
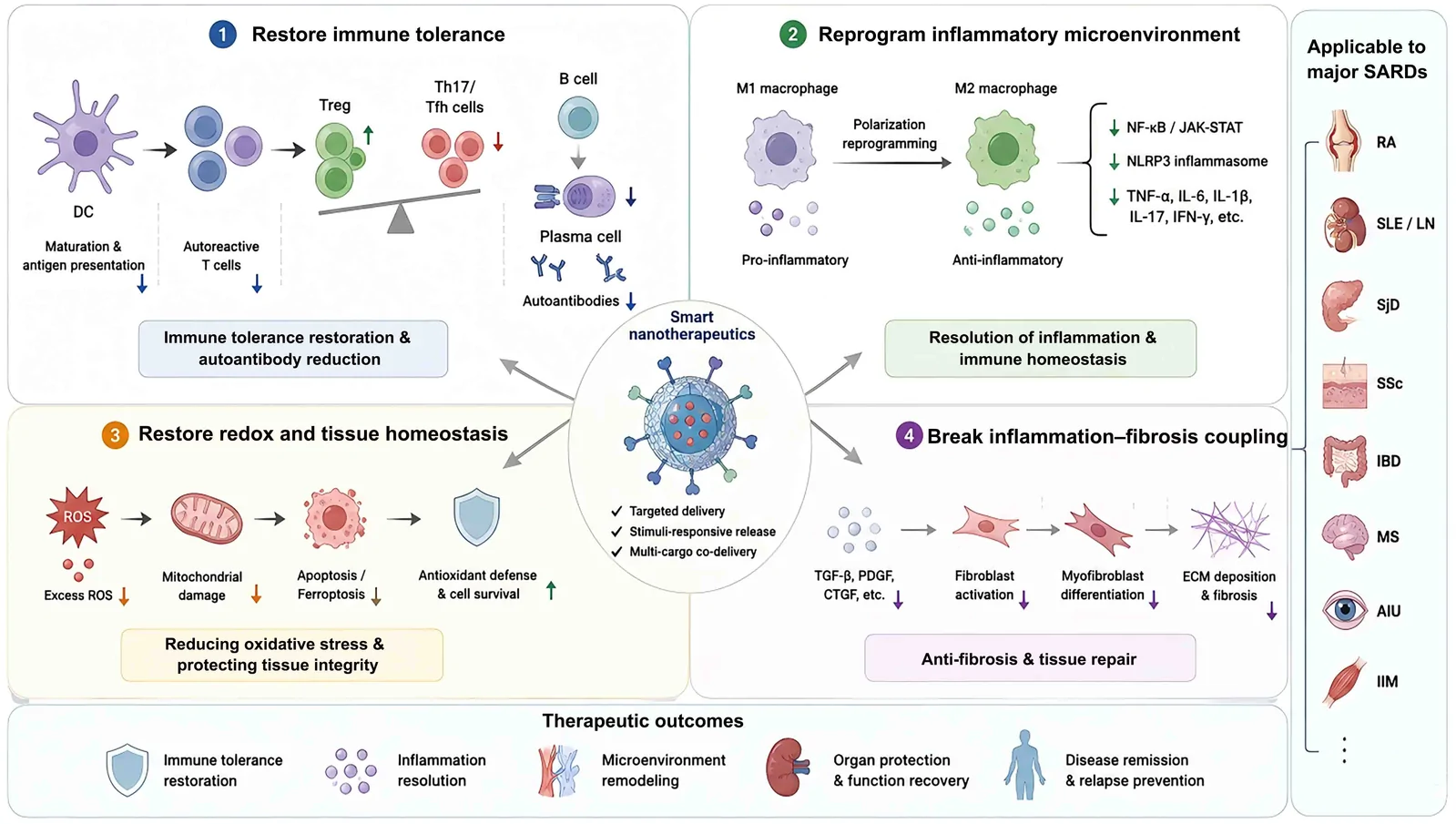
Multilevel mechanisms of nanotherapeutic intervention in rheumatoid arthritis and other systemic autoimmune rheumatic diseases. Rheumatoid arthritis (RA), for which the preclinical nanomedicine literature is comparatively well developed, provides a mechanistic anchor illustrating how nano-enabled delivery can intersect with nuclear factor kappa B (NF-κB)/Janus kinase–signal transducer and activator of transcription (JAK–STAT) signaling, NLRP3 inflammasome activation, inflammatory cytokine production, oxidative stress, immune-tolerance pathways, and inflammation–fibrosis coupling. These mechanisms are not assumed to operate identically across SARDs and require disease- and organ-specific validation. The M1/M2 labels are retained only as simplified functional extremes used in many preclinical studies; *in vivo* macrophages comprise heterogeneous, overlapping states that should be resolved using multi-marker, single-cell, spatial, and functional analyses. Antifibrotic activity likewise requires pathway and functional evidence beyond reduced collagen deposition alone. AIU, autoimmune uveitis; CTGF, connective tissue growth factor; DC, dendritic cell; ECM, extracellular matrix; IBD, inflammatory bowel disease; IFN, interferon; IIM, idiopathic inflammatory myopathy; IL, interleukin; JAK, Janus kinase; LN, lupus nephritis; M1, classically activated macrophage phenotype; M2, alternatively activated macrophage phenotype; MS, multiple sclerosis; NF-κB, nuclear factor kappa B; NLRP3, NOD-like receptor family pyrin domain-containing 3; PDGF, platelet-derived growth factor; RA, rheumatoid arthritis; ROS, reactive oxygen species; SARDs, systemic autoimmune rheumatic diseases; SjD, Sjögren’s disease; SLE, systemic lupus erythematosus; SSc, systemic sclerosis; STAT, signal transducer and activator of transcription; Tfh, T follicular helper cell; TGF-β, transforming growth factor beta; Th17, T helper 17 cell; TNF, tumor necrosis factor; Treg, regulatory T cell.

**Figure 4:**
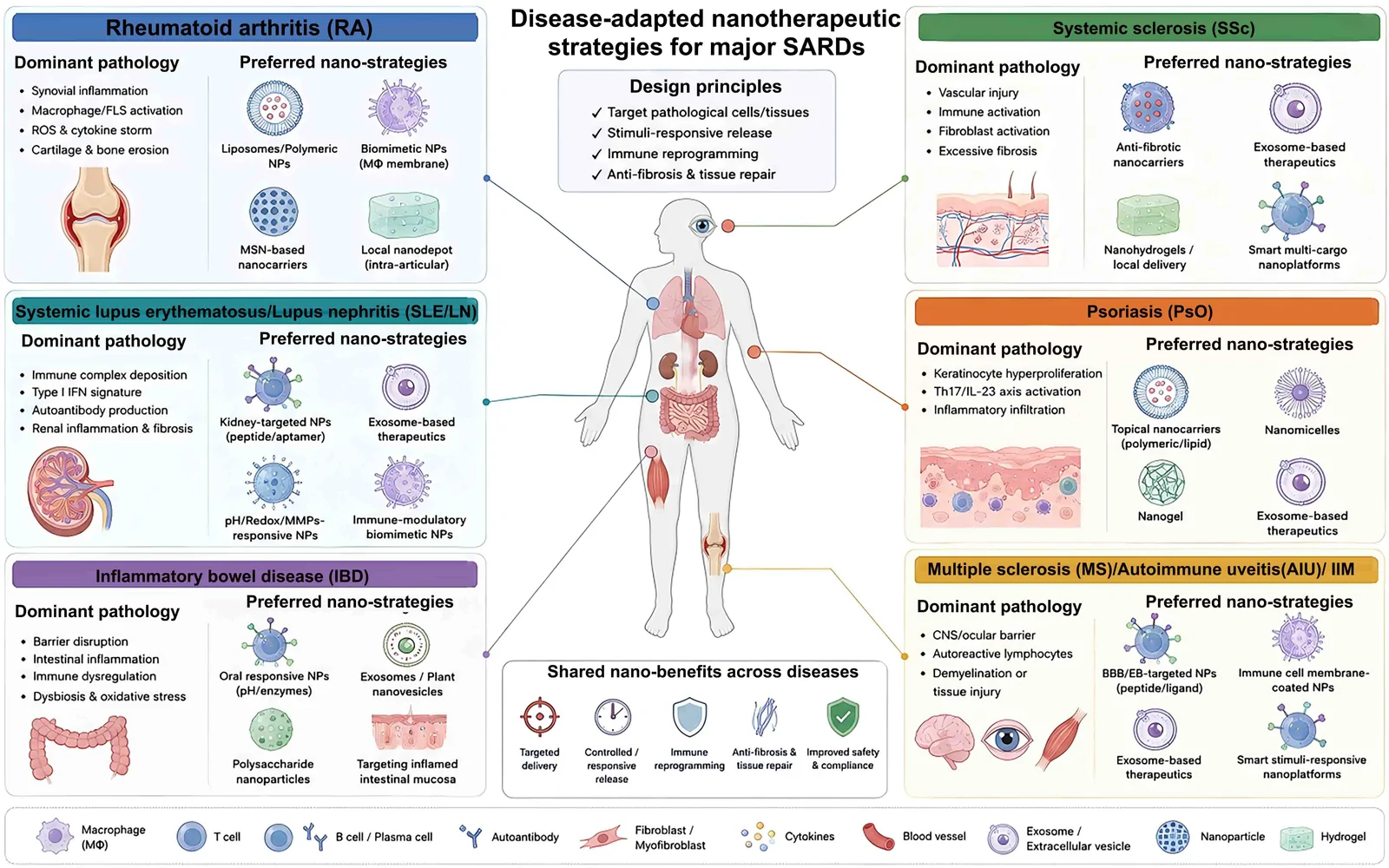
Disease-adapted nanotherapeutic strategies in core systemic autoimmune rheumatic diseases and transferable immune-mediated disease contexts. The RA, SLE/LN, SSc, and IIM components represent diseases within the primary scope of this Review; SjD is discussed in the main text where disease-specific nanomedicine evidence remains limited. Psoriasis, inflammatory bowel disease, multiple sclerosis, and autoimmune uveitis are shown only as transferable engineering contexts—for example, barrier penetration, local delivery, or stimulus-responsive design—and are not interpreted as direct evidence of SARD efficacy. RA serves as the mechanistic anchor, illustrating synovial exposure, cell/tissue engagement, controlled release, and modulation of NF-κB, NLRP3, MAPK, and redox pathways. Each design principle requires independent revalidation in the relevant disease, organ, and therapeutic context. AIU, autoimmune uveitis; BBB, blood–brain barrier; CNS, central nervous system; EB, endothelial barrier; FLS, fibroblast-like synoviocyte; IBD, inflammatory bowel disease; IFN, interferon; IIM, idiopathic inflammatory myopathy; IL-23, interleukin-23; LN, lupus nephritis; MMPs, matrix metalloproteinases; MSN, mesoporous silica nanoparticle; MS, multiple sclerosis; MΦ, macrophage; NP, nanoparticle; NPs, nanoparticles; PsO, psoriasis; RA, rheumatoid arthritis; ROS, reactive oxygen species; SARDs, systemic autoimmune rheumatic diseases; SjD, Sjögren’s disease; SLE, systemic lupus erythematosus; SSc, systemic sclerosis; Th17, T helper 17 cell.

### 3.1 Rheumatoid arthritis: the best-developed preclinical evidence base for disease-adapted nanotherapy

RA is particularly amenable to nanotherapeutic testing because inflamed synovium combines vascular activation, macrophage and fibroblast-like synoviocyte (FLS) expansion, oxidative stress, cytokine amplification, and progressive osteochondral damage. These features create opportunities for prolonged joint exposure and cell-directed delivery, but they also create a recurrent interpretive problem: high joint fluorescence may reflect vascular permeability and phagocytic sequestration rather than receptor-specific targeting. Quantitative biodistribution, cell-resolved uptake, and ligand-dependence should therefore accompany claims of precision delivery.

#### 3.1.1 Delivery, biodistribution, and target engagement

Recent RA platforms illustrate a shift from simple accumulation toward more explicit targeting logic. Monocyte-membrane-coated drug nanocrystals co-delivering the NOD-like receptor family pyrin domain-containing 3 (NLRP3) inflammasome inhibitor MCC950 and low-dose dexamethasone showed inflammatory tropism and reduced arthritis severity in adjuvant-induced arthritis, while a 2026 dual-ligand nanocapsule targeted activated endothelium and inflammatory macrophages through P-selectin- and integrin-associated interactions, respectively (31, 35). A thermoresponsive magnetic methotrexate nanocarrier further combined external magnetic guidance with near-infrared-triggered release (36). These studies are informative because they couple localization with a defined delivery mechanism; nevertheless, translation will require absolute or dose-normalized tissue exposure, uptake in pathogenic versus homeostatic cell subsets, and receptor-blocking or competition experiments when ‘active targeting’ is claimed.

#### 3.1.2 Macrophage and stromal reprogramming beyond the M1/M2 dichotomy

Many RA nanotherapy studies describe efficacy as conversion of “M1” to “M2” macrophages. This terminology remains a convenient shorthand for pro-inflammatory versus inflammation-resolving tendencies in reductionist models, but it should not be interpreted as a binary *in vivo* taxonomy. Single-cell and spatial studies have identified multiple synovial macrophage states with distinct inflammatory, tissue-resident, remodeling, and remission-associated programs. Nine transcriptionally distinct synovial macrophage clusters have been described in human RA, and recent synthesis across rheumatic diseases emphasizes a continuum that includes mixed and disease-stage-specific phenotypes rather than two stable endpoints (37, 38). Accordingly, future nanotherapy studies should report marker panels, transcriptional states, metabolic features, spatial localization, and functional outputs rather than relying on one or two M1/M2 markers. Preserving protective MerTK-positive tissue-resident macrophage programs may be as important as suppressing pathogenic inflammatory subsets.

#### 3.1.3 Nucleic-acid delivery and pathway-level evidence

Nucleic-acid nanotherapy offers the possibility of intervening upstream of cytokine output. The fluorinated PAMAM/miR-23b system is a useful example because it linked delivery to reduced TAB2, TAB3, and IKKα expression and lower inflammatory cytokine production (25). These data support involvement of the NF-κB axis, but the mechanistic strength of future studies would increase with direct measurement of pathway activation, such as IκB degradation, p65 phosphorylation and nuclear translocation, chromatin-level target engagement, or rescue experiments. This distinction between pathway-consistent downstream effects and direct pathway validation should be maintained throughout the field.

#### 3.1.4 Microenvironment-responsive release: physiological relevance matters

Acidic pH, elevated ROS, and protease activity can be exploited for RA-responsive release, but responsiveness is not synonymous with disease specificity. Synovial pH and oxidant burden vary with disease activity, vascularity, oxygen tension, medication, and sampling compartment. A persuasive responsive system should therefore be tested in clinically plausible ranges, relevant synovial fluid or protein-containing media, and appropriate diseased versus non-diseased controls. Trigger-insensitive formulations and measurements of premature release in plasma are particularly important. Systems that require extreme acidity, very high hydrogen peroxide concentrations, or externally applied energy may demonstrate engineering feasibility without establishing autonomous responsiveness in human RA.

### 3.2 Systemic lupus erythematosus and lupus nephritis: from systemic immune modulation to renal compartment targeting

SLE illustrates both the promise and difficulty of nanomedicine in a multisystem autoimmune disease. A platform that improves exposure in the kidney may not address pathogenic activity in skin, vasculature, or the central nervous system, whereas a broadly immunosuppressive nanoparticle may reproduce the systemic liabilities of its free drug. Disease-appropriate design therefore requires a clear definition of whether the intended objective is systemic immune reprogramming, renal delivery, immune-complex interception, antioxidant protection, or restoration of tolerance.

Biomimetic delivery has shown measurable pharmacokinetic advantages. RBC membrane-coated cyclosporine-loaded PLGA nanoparticles increased cyclosporine half-life and AUC in lupus-prone mice, but those quantitative effects should not be generalized to other membrane systems or diseases (30). More recent work has moved toward mechanistically richer LN platforms. Engineered mesenchymal stromal cell-derived exosomes integrating ceria-mediated ROS regulation with rapamycin delivery accumulated in inflamed kidneys and produced combined antioxidant, immunosuppressive, and reparative effects in MRL/lpr mice (39). In 2026, a collagen IV-α3-binding liposomal system was reported to localize to glomeruli and deliver prednisolone with improved renal functional and histological outcomes, providing a useful example of anatomically defined target engagement rather than nonspecific ‘kidney targeting’ (40).

For LN, future studies should quantify glomerular, tubular, interstitial, and immune-cell exposure separately where possible. Fluorescent renal accumulation alone can be misleading because nanoparticles may be retained in phagocytes or cleared through renal pathways without reaching the therapeutic compartment. Mechanistic endpoints should include renal immune-cell composition, autoantibody or immune-complex biology, complement activity where relevant, fibrosis pathways, and renal function—not histology alone. Because lupus therapies are usually chronic and combinatorial, repeated dosing and interaction with background immunosuppression are central translational requirements.

### 3.3 Systemic sclerosis: distinguishing true antifibrotic activity from reduced collagen staining

SSc requires a different evidentiary framework because vascular injury, immune activation, fibroblast state transitions, extracellular-matrix remodeling, and organ-specific fibrosis evolve over time. A reduction in total collagen or dermal thickness after treatment is supportive but does not by itself demonstrate direct modulation of fibrosis. Stronger mechanistic evidence includes reduced myofibroblast differentiation, altered transforming growth factor beta (TGF-β)/SMAD signaling, suppression of profibrotic transcriptional programs, changes in matrix synthesis and degradation, tissue stiffness, and functional organ outcomes.

Recent nanotherapeutic studies illustrate this higher standard. A20 mRNA delivered with lipid nanoparticles suppressed TRAF6/NF-κB signaling, reduced DREAM expression, inhibited SMAD2 activation, and attenuated skin and lung fibrosis in a bleomycin model, linking molecular pathway changes to fibrotic outcomes (41). A 2026 bioengineered exosome–liposome platform carrying kaempferol likewise combined antioxidant effects with inhibition of TGF-β/SMAD signaling and myofibroblast activation in experimental scleroderma (42). Such studies are more mechanistically informative than collagen reduction alone, although the bleomycin model remains an imperfect surrogate for the vascular, immune, and chronic multiorgan heterogeneity of human SSc.

### 3.4 Sjögren’s disease and idiopathic inflammatory myopathies: important gaps in disease-specific evidence

Compared with RA, SLE/LN, and SSc, the disease-specific experimental nanotherapy literature in SjD and IIM is limited and cannot support a mature catalogue of validated platforms. For SjD, glandular epithelial injury, altered local immune niches, and secretory dysfunction motivate hypotheses involving gland retention or cell-selective cargo delivery; for IIM, heterogeneity across inflammatory myopathy subtypes and muscle-targeting barriers suggest different therapeutic design problems. Nevertheless, the gland-directed carriers, immunomodulatory EV approaches, muscle-directed carriers, and nucleic-acid delivery concepts identified in **Table 2** are explicitly proposed applications unless a disease-specific study is cited. They should not be interpreted as demonstrated SjD or IIM therapeutic efficacy. Appropriate next steps include model qualification, quantitative delivery studies, and disease-relevant salivary or muscle functional readouts.

**Table 2:** Evidence-status-stratified nanotherapeutic strategies and proposed applications across core SARDs.

Disease	Dominant pathological features	Evidence status and disease-specific nanotherapeutic strategies	Principal cellular/molecular targets	Main therapeutic functions	Key translational considerations
Rheumatoid arthritis (RA)	Persistent synovitis; activated endothelium; heterogeneous macrophage states; fibroblast-like synoviocyte (FLS) expansion; oxidative stress; cartilage and bone damage	DEMONSTRATED IN RA MODELS: membrane/ligand-engineered and nucleic-acid nanocarriers, selected stimuli-responsive systems, and local depots (Refs. 25, 31, 34–36). Evidence varies by formulation and endpoint.	Activated endothelium; pathogenic macrophage states; FLS; NF-κB/NLRP3/MAPK/redox networks	Increase synovial exposure; suppress inflammatory and stromal activation; reduce cytokine/inflammasome signaling; protect cartilage and bone	Quantitative joint and cell-subset biodistribution; receptor/target-engagement controls; chronic arthritis models; repeated-dose safety
Systemic lupus erythematosus/lupus nephritis (SLE/LN)	Loss of tolerance; autoreactive lymphocytes; autoantibodies and immune complexes; interferon/inflammatory signaling; glomerular and tubulointerstitial injury	DEMONSTRATED IN SLE/LN MODELS: biomimetic carriers, renal/glomerular-directed liposomes and engineered EV formulations (Refs. 30, 39, 40); not validated across all lupus manifestations.	Renal immune compartments; glomerular targets; Treg/Tfh/B-cell networks; ROS and inflammatory pathways	Improve renal or systemic drug exposure; reduce immune-complex-associated and oxidative injury; restore immune regulation; preserve renal function	Compartment-resolved renal biodistribution; glomerular target engagement; systemic-versus-renal efficacy; background immunosuppression; repeated-dose immune safety
Systemic sclerosis (SSc)	Vascular injury; immune activation; fibroblast and myofibroblast state transitions; extracellular-matrix accumulation; organ-specific fibrosis	DEMONSTRATED IN SSc MODELS: selected mRNA-LNP and engineered EV platforms with antifibrotic readouts (Refs. 41, 42). Local hydrogels and microneedles remain investigational extensions.	TRAF6/NF-κB; TGF-β/SMAD; fibroblast-state transitions; oxidative stress; immune–stromal coupling	Suppress inflammatory–fibrotic coupling and myofibroblast activation; reduce matrix synthesis; support tissue repair and organ function	Mechanistic antifibrotic endpoints beyond collagen; skin plus internal-organ models; tissue function; long-term accumulation and safety
Sjögren’s disease (SjD)	Glandular epithelial injury; B-cell-rich immune niches; interferon activity; salivary/lacrimal barrier dysfunction	PROPOSED/NOT ESTABLISHED IN SjD: gland-retentive or gland-targeted carriers, immunomodulatory EVs, and responsive-release concepts extrapolated from gland biology or other diseases; no disease-specific validation is claimed here.	Candidate targets (proposed): epithelial–immune crosstalk; B-/T-cell niches; gland inflammatory signals.	Potential objectives (not demonstrated outcomes): reduce gland inflammation and preserve or recover secretory function.	Evidence gap: proposed designs require disease-specific biodistribution, secretion and gland-function assays, and local safety testing before efficacy can be inferred.
Idiopathic inflammatory myopathies (IIM)	Subtype-specific immune dysregulation; myofiber injury; interferon signatures; muscle inflammation and damage	PROPOSED/NOT ESTABLISHED IN IIM: muscle-directed nanocarriers, nucleic-acid delivery and local anti-inflammatory concepts motivated by myositis biology; no disease-specific experimental efficacy is cited for these proposals.	Candidate targets (proposed): muscle-resident immune cells; myofibers; interferon-related and subtype-specific pathways.	Potential objectives (not demonstrated outcomes): reduce subtype-specific muscle inflammation and improve muscle function.	Evidence gap: proposed designs require myositis subtype stratification, muscle biodistribution and functional endpoints before efficacy can be inferred.

Evidence-status note: “DEMONSTRATED” indicates selected published disease-specific preclinical examples (not clinical efficacy), whereas “PROPOSED” indicates hypothesis-generating designs without disease-specific validation cited in this Review. Targets and potential functions listed for SjD and IIM are research objectives, not demonstrated treatment effects. Abbreviations: EV, extracellular vesicle; FLS, fibroblast-like synoviocyte; IIM, idiopathic inflammatory myopathy; LN, lupus nephritis; LNP, lipid nanoparticle; MAPK, mitogen-activated protein kinase; NF-κB, nuclear factor kappa B; NLRP3, NOD-like receptor family pyrin domain-containing 3; RA, rheumatoid arthritis; ROS, reactive oxygen species; SARDs, systemic autoimmune rheumatic diseases; SjD, Sjögren’s disease; SLE, systemic lupus erythematosus; SSc, systemic sclerosis; Tfh, T follicular helper cell; TGF-β, transforming growth factor beta; TRAF6, tumor necrosis factor receptor-associated factor 6; Treg, regulatory T cell.

### 3.5 Transferable evidence from adjacent immune-mediated diseases

Studies in osteoarthritis, gout, inflammatory bowel disease, psoriasis, multiple sclerosis, and autoimmune uveitis can still provide valuable engineering lessons—for example, cartilage penetration, mucosal delivery, blood–brain or ocular barrier transport, local depot design, and antigen-specific tolerance. In this Review, however, such studies are treated as supportive engineering evidence rather than direct proof of efficacy in SARDs. Disease-specific pathology, tissue barriers, immune-cell composition, and dosing requirements must be revalidated before these design principles are extrapolated to RA, SLE/LN, SSc, SjD, or IIM.

### 3.6 Cross-cutting interpretation of preclinical efficacy

Interpretation of preclinical efficacy should be tailored to the mechanistic claims being made. Where improved delivery is claimed, a free-cargo comparator can establish the incremental formulation effect; where intrinsic carrier activity is plausible, an unloaded-carrier comparator is informative. Receptor-dependence or competition controls are relevant when specific targeting is asserted, whereas trigger-insensitive formulations are appropriate when stimulus-dependent release is central to the therapeutic hypothesis. Not every study requires the complete set of sophisticated assays: the choice of comparators and readouts should follow platform composition, route, therapeutic cargo, and the causal question. When feasible, quantitative exposure and disease-relevant functional readouts should complement efficacy phenotypes.

## 4 Discussion, core challenges, and prospective outlook

### 4.1 From proof-of-concept nanodelivery to evidence-based precision nanotherapy

The central question for SARD nanomedicine is no longer whether nanoparticles can reduce inflammation in a rodent model; many platforms can. The more consequential question is whether nanoengineering creates a reproducible therapeutic advantage that cannot be achieved by conventional formulation at an acceptable level of complexity, toxicity, and cost. Demonstrating that advantage requires mechanistic deconvolution, disease-relevant pharmacology, and longitudinal safety rather than increasingly elaborate material design alone. The major translational barriers and next-generation design priorities are summarized in **Figure 5**. As a focused disease-specific synthesis, **Figure 6** illustrates how nanomaterial-enabled therapy in RA can connect synovial microenvironmental features with modulation of macrophages, fibroblast-like synoviocytes, lymphocytes, osteoclasts, and key inflammatory signaling pathways.

**Figure 5:**
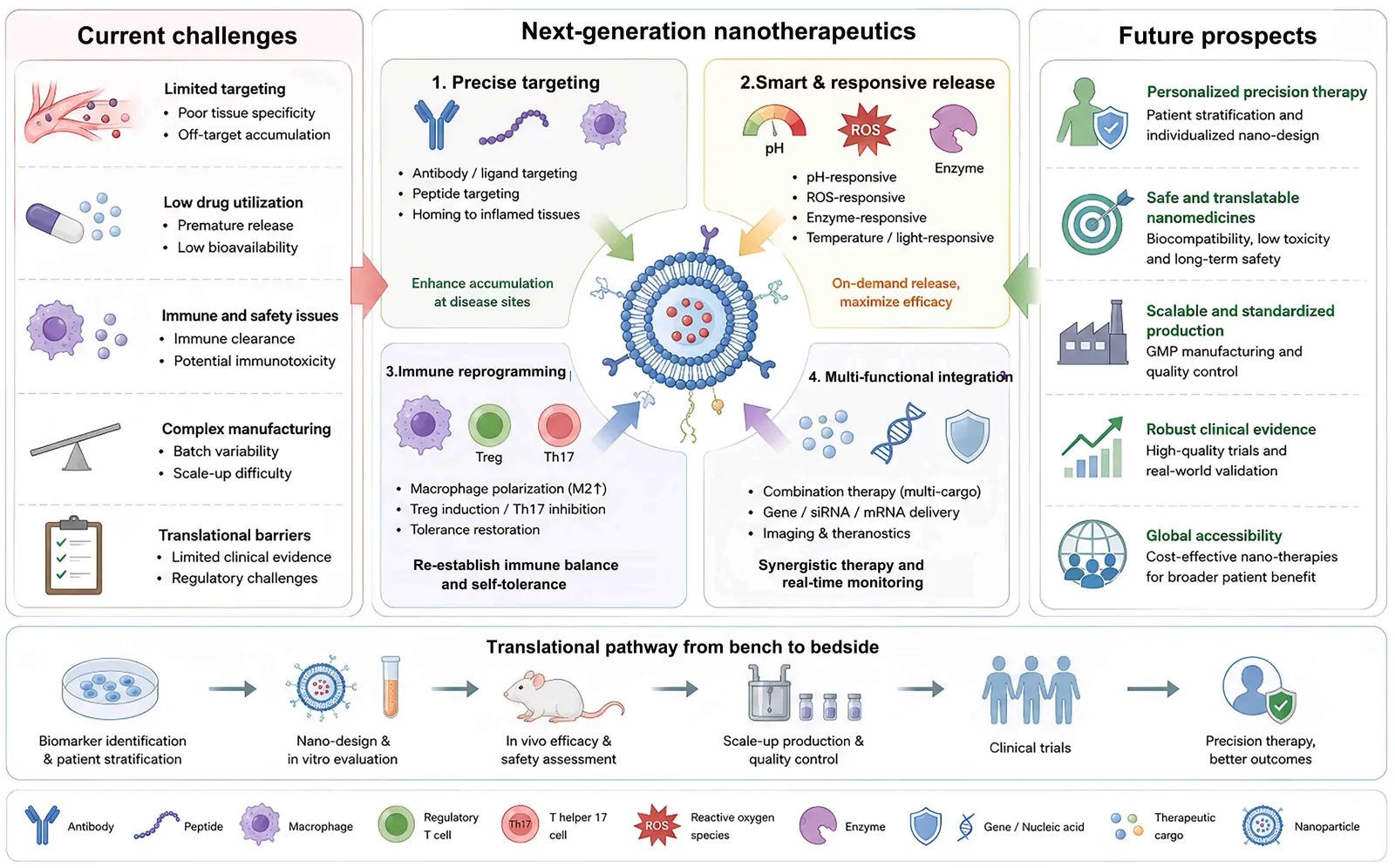
Translational challenges, next-generation design strategies, and future directions for nanotherapeutics in systemic autoimmune rheumatic diseases. Major barriers include insufficient disease and tissue specificity, off-target accumulation, premature or physiologically unvalidated release, immune clearance and immunotoxicity, repeated-dose accumulation, manufacturing variability, limited clinical evidence, and regulatory complexity. Next-generation systems should therefore prioritize four linked principles: (1) quantitatively validated targeting and target engagement rather than localization alone; (2) responsive release calibrated to measured disease-relevant pH, ROS, enzyme, temperature, or other trigger ranges; (3) cell-state-resolved immune modulation rather than binary M1/M2 assumptions; and (4) simplified, scalable, quality-controlled formulation design. Depending on material persistence, cargo, route, and intended dosing regimen, risk-based safety assessment may include toxicokinetics, organ clearance, immunogenicity, degradation products, and effects on protective immune function. These considerations are complementary and product-specific rather than a mandatory linear development sequence. GMP, good manufacturing practice; M2, alternatively activated macrophage phenotype; mRNA, messenger RNA; pH, hydrogen ion potential; ROS, reactive oxygen species; SARDs, systemic autoimmune rheumatic diseases; siRNA, small interfering RNA; Th17, T helper 17 cell; Treg, regulatory T cell.

**Figure 6:**
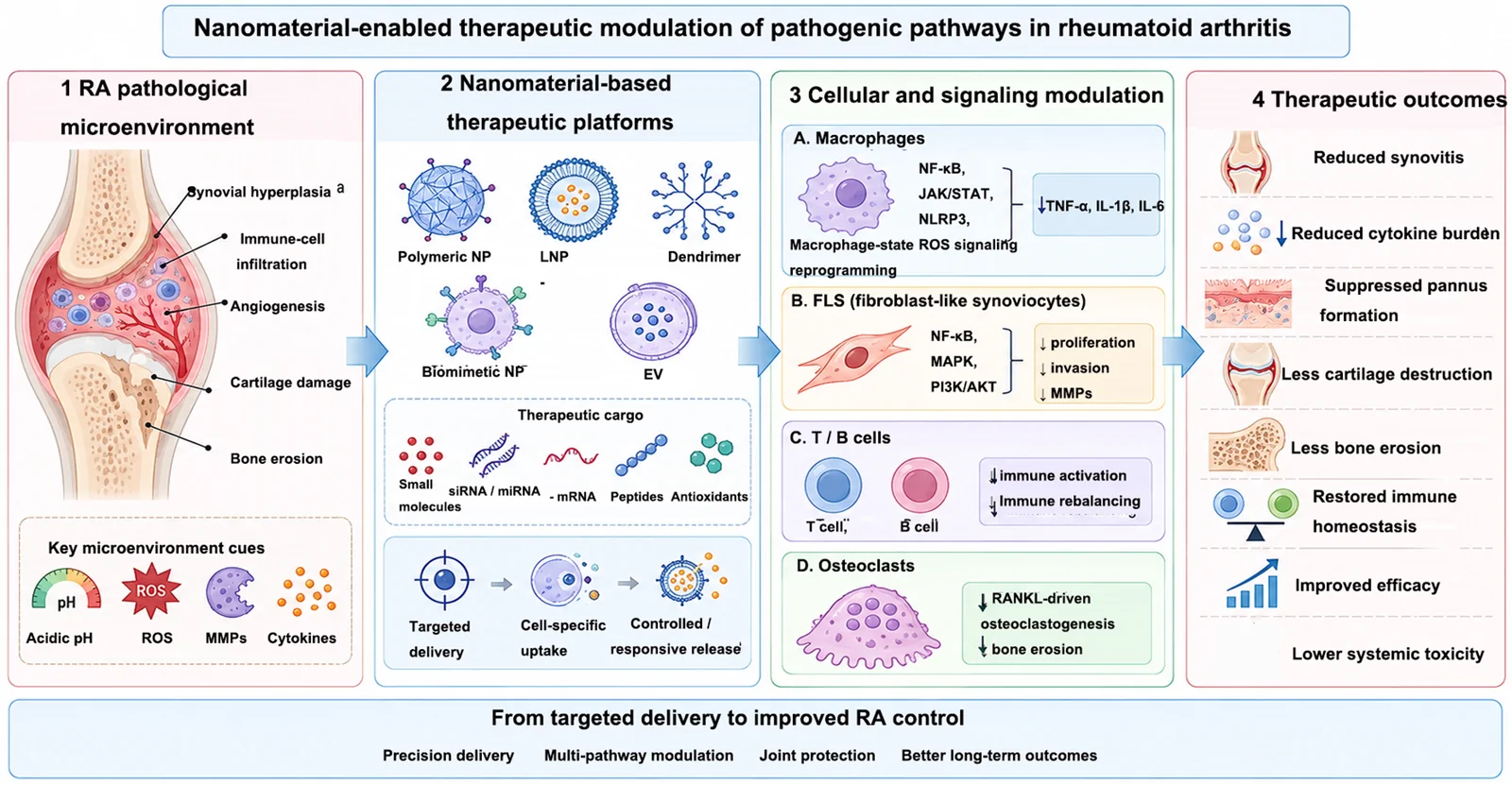
Nanomaterial-enabled therapeutic modulation of pathogenic pathways in rheumatoid arthritis. Nanomaterial-based platforms, including polymeric nanoparticles, lipid nanoparticles (LNPs), dendrimers, biomimetic nanoparticles, and extracellular vesicles (EVs), can enhance delivery of therapeutic cargos to inflamed synovial tissue in rheumatoid arthritis (RA) through formulation-dependent increases in local exposure, cell-specific uptake, and controlled or stimuli-responsive release. These systems have been designed to modulate pathogenic programs in synovial macrophages, fibroblast-like synoviocytes (FLSs), T and B lymphocytes, and osteoclasts, including nuclear factor kappa B (NF-κB), Janus kinase/signal transducer and activator of transcription (JAK/STAT), NOD-like receptor family pyrin domain-containing 3 (NLRP3) inflammasome, mitogen-activated protein kinase (MAPK), phosphoinositide 3-kinase/protein kinase B (PI3K/AKT), and redox-related signaling. The resulting effects may include reduced pro-inflammatory cytokine production, reprogramming of pathogenic macrophage states, suppression of FLS proliferation and invasion and matrix metalloproteinase (MMP) production, inhibition of receptor activator of nuclear factor-κB ligand (RANKL)-driven osteoclastogenesis, and restoration of immune homeostasis. Collectively, these mechanisms can attenuate synovitis, pannus formation, cartilage destruction, and bone erosion while potentially improving therapeutic efficacy and reducing systemic exposure or toxicity. The schematic is mechanistic and does not imply that every platform engages all pathways or achieves equivalent targeting *in vivo*. AKT, protein kinase B; EV, extracellular vesicle; FLS, fibroblast-like synoviocyte; IL, interleukin; JAK, Janus kinase; LNP, lipid nanoparticle; MAPK, mitogen-activated protein kinase; MMP, matrix metalloproteinase; NF-κB, nuclear factor kappa B; NLRP3, NOD-like receptor family pyrin domain-containing 3; NP, nanoparticle; PI3K, phosphoinositide 3-kinase; RA, rheumatoid arthritis; RANKL, receptor activator of nuclear factor-κB ligand; ROS, reactive oxygen species; STAT, signal transducer and activator of transcription; TNF, tumor necrosis factor.

Three claims deserve particular caution. First, accumulation is not equivalent to targeting. A long-circulating formulation can accumulate in inflamed tissue without engaging the intended cell or receptor. Second, response to an artificial trigger is not equivalent to disease specificity. pH, ROS, and MMP activity are continuous biological variables with substantial overlap between diseased and healthy tissues. Third, improved efficacy of a loaded nanoparticle is not automatically attributable to the cargo, because carriers may have antioxidant, anti-inflammatory, membrane-interactive, or immunomodulatory activity. These distinctions should shape both experimental design and language used to describe results.

### 4.2 Heterogeneity of the SARD microenvironment limits universal ‘smart-release’ assumptions

Acidic pH, oxidative stress, and increased protease activity are often presented as generic hallmarks of SARD lesions, but this is an oversimplification. RA synovium can be acidic and oxidatively stressed, whereas the relevant microenvironment in LN differs between glomerular, tubulointerstitial, vascular, and immune-cell compartments. SSc evolves from inflammatory and vascular stages toward organ-specific fibrotic niches, and SjD or IIM presents different glandular or muscle microenvironments. Even within one disease, treatment, disease duration, perfusion, hypoxia, infection, and tissue damage can change the magnitude of these cues.

Responsive nanomaterials should therefore be calibrated against measured pathophysiological ranges rather than convenient laboratory conditions. Release kinetics should be tested in plasma and disease-relevant biofluids, across overlapping normal and pathological trigger ranges, and under repeated exposure. A system that releases cargo at pH 5.0 may function efficiently in an endosome but should not be described as synovial-acidity-specific unless extracellular disease-site conditions support that claim. Similar caution applies to ROS-sensitive linkers tested with supraphysiological peroxide concentrations and MMP-responsive systems evaluated with purified enzymes at activities not demonstrated in the target tissue.

### 4.3 Macrophage targeting should move beyond the binary M1/M2 framework

The M1/M2 framework remains useful as a simplified language for *in vitro* polarization, but contemporary human tissue studies show that macrophages in rheumatic disease occupy a continuum of transcriptional, metabolic, spatial, and functional states. In RA, inflammatory, remodeling, resident barrier, osteoclastogenic, and remission-associated macrophage populations coexist; in SSc and LN, macrophage states can be simultaneously inflammatory and profibrotic (37, 38). A therapeutic strategy designed simply to ‘convert M1 to M2’ may therefore suppress one readout while leaving pathogenic macrophage programs intact or even promoting fibrosis in another tissue.

Future studies should define the macrophage state being targeted, its anatomical niche, and the functional endpoint expected to change. Single-cell RNA sequencing, spatial transcriptomics, multiplex imaging, and functional assays can establish whether a nanotherapy depletes, reprograms, or redistributes a pathogenic subset. At minimum, multi-marker panels and functional cytokine, phagocytic, metabolic, or matrix-remodeling readouts should complement canonical M1/M2 markers.

### 4.4 Antifibrotic claims require pathway and function, not collagen alone

Fibrosis is a dynamic process involving fibroblast activation, myofibroblast differentiation, matrix production and crosslinking, altered matrix degradation, mechanotransduction, vascular dysfunction, and immune–stromal crosstalk. Reduced collagen staining after treatment can result indirectly from less inflammation or tissue injury and does not prove direct antifibrotic action. Strong evidence should combine histology with pathway-level and functional endpoints: TGF-β/SMAD activity, ACTA2/α-SMA and fibroblast-state markers, matrix synthesis and turnover, tissue stiffness, hydroxyproline or quantitative collagen assays, relevant organ physiology, and ideally intervention or rescue experiments that establish causality. Recent A20 mRNA-LNP and bioengineered extracellular-vesicle studies in experimental SSc exemplify this more mechanistic approach (41, 42).

### 4.5 Biodistribution, target engagement, and the carrier-versus-cargo problem

A central translational weakness is that biodistribution is often presented qualitatively and target engagement is inferred from organ fluorescence. Quantitative mass balance, dose-normalized tissue concentration, pharmacokinetic modeling, and cell-subset-resolved uptake are needed to determine where the administered dose actually goes. Modern nanomedicine research also emphasizes that high avidity does not guarantee improved whole-organ biodistribution and that liver, spleen, and mononuclear phagocyte uptake remain dominant barriers for many systemic formulations (43).

The most informative controls depend on the causal claim and can be selected using the decision logic outlined in Section 3.6. For example, a ceria-based drug carrier may require separation of catalytic carrier activity from cargo pharmacology, while a membrane-coated or extracellular-vesicle formulation may require source-matched and processing-matched controls. In studies claiming active homing, receptor competition or functional perturbation can be considered when a tractable receptor is known; in responsive-delivery studies, a matched trigger-insensitive formulation is often more informative. These are context-sensitive analytical options, not an obligatory universal battery, and should be prioritized according to feasibility, product complexity, and the intended mechanism.

### 4.6 Chronic-disease biosafety: platform-specific risks and longitudinal evaluation

Because many SARDs require prolonged treatment, the duration and intensity of safety testing should reflect the intended dosing regimen, material persistence, patient population, and administration route. Short-term normal histology alone cannot establish chronic safety, but repeated dosing is not an identical or mandatory test for every early-stage material. Well-recognized nanomedicine immunotoxicity concerns include certain complement-mediated infusion reactions and formulation-specific inflammatory responses; their frequency and mechanisms vary widely among nanomaterial classes (44, 45). For polyethylene glycol (PEG)-containing formulations, anti-PEG antibodies and accelerated blood clearance are documented considerations in particular experimental and clinical settings, not a property that can be extrapolated to all carriers (46). Inorganic materials with limited degradation may warrant longer-term retention and organ-clearance assessments, whereas biodegradable polymeric or lipid formulations require characterization of their actual breakdown products and exposure kinetics. Biological coatings and extracellular vesicles introduce source, co-isolated component, identity, and batch-consistency uncertainties; standardized characterization and functional testing are especially important before asserting reproducible immune compatibility (47).

Safety evaluation should distinguish carrier-associated toxicity from cargo pharmacology and from the baseline hazards of concomitant immunosuppressive treatment. Depending on route, exposure, and material class, relevant assessments may include repeat-exposure toxicokinetics, organ retention and recovery, anti-carrier antibodies, complement/cytokine responses, hematology, relevant immune-cell functions, infection susceptibility, and degradation-product analysis; these tests should be selected on a risk-based rather than universal-checklist basis (44–47). Disease-associated changes in serum proteins, vascular barriers, mononuclear-phagocyte function, and renal or hepatic physiology can alter nanoparticle disposition, although direct evidence remains uneven across individual SARDs and formulations (48). Disease-model comparisons should therefore be used where biologically justified, and observations from cancer, infection, or other pathological contexts should not be presented as established pharmacokinetic effects in RA, LN, or SSc without confirmatory studies.

### 4.7 Regulatory and manufacturing hurdles should shape design early

Regulatory development should be integrated early into formulation design, while distinguishing general quality principles from product-specific expectations. US Food and Drug Administration (FDA) guidance addresses characterization, manufacturing controls, stability, and the relationship between nanomaterial attributes and product performance; as guidance, it communicates current agency thinking and is not itself a universally binding checklist (49). The European Medicines Agency/Heads of Medicines Agencies 2025 horizon-scanning report identifies emerging classification, analytical, and assessment questions but is not equivalent to a legally binding authorization standard (50). Applicable requirements depend on jurisdiction, product classification (for example, drug, biological product, or combination product), clinical indication, route, and material composition. Early engagement with the relevant authority may help clarify an appropriate evidence package for complex biomimetic, extracellular-vesicle, or responsive platforms.

Critical quality attributes and test methods should be selected through a product-specific risk assessment rather than copied from a universal list. Depending on the formulation and intended use, relevant attributes may include particle-size distribution, morphology, surface composition and charge, encapsulation/loading, release kinetics, aggregation, residual impurities, biological potency, microbial control/sterility, and stability. A property may be critical for one carrier yet of limited relevance for another; for example, release testing differs between a freely bioactive inorganic nanozyme and a drug-loaded degradable polymer, while donor/source qualification and identity/potency assays are especially relevant for biological coatings and extracellular vesicles. Analytical procedures, acceptance criteria, scale-up comparability, and good manufacturing practice controls should follow the specific product’s quality-risk profile and the regulatory framework of the intended jurisdiction (49, 50). The main cross-platform translational barriers and context-dependent validation priorities are summarized in **Table 3**.

**Table 3:** Key translational barriers and priority solutions for next-generation nanotherapeutics.

Translational barrier	Current limitation	Potential clinical consequence	Priority validation	Recommended next-generation solution
Insufficient disease and tissue specificity	Passive accumulation, vascular leak and phagocytic sequestration can be mislabeled as active targeting; target engagement is often not directly demonstrated.	Off-target exposure, incomplete efficacy and disruption of normal immune function	Absolute and dose-normalized biodistribution; cell-subset uptake; receptor-dependence/competition; target-engagement biomarkers.	Use disease- and stage-adapted ligands only when the target is validated in human tissue; distinguish accumulation from engagement.
Premature or poorly controlled drug release	Responsive release is often validated in simplified buffers or with trigger levels outside physiological/pathological ranges.	Low therapeutic index, dose escalation and systemic toxicity	Release in plasma and disease-relevant biofluids; overlapping normal/pathological pH, ROS or enzyme ranges; trigger-insensitive controls.	Calibrate triggers to measured human disease conditions and report premature leakage and on-target release quantitatively.
Long-term biocompatibility and accumulation	Repeated administration, cumulative organ exposure, immunogenicity, complement activation, degradation products and effects on normal immune function remain insufficiently characterized.	Delayed organ toxicity, chronic inflammation or immune dysfunction during long-term treatment	Risk-based toxicokinetics and organ-clearance assessment; targeted immunogenicity/complement testing where justified; degradation and recovery measures matched to material, cargo, route, and dosing schedule.	Design for material-specific clearance and characterize products of degradation; select longitudinal safety studies according to intended use and exposure.
Model-to-human disease mismatch	Short-term induced animal models often fail to reproduce patient heterogeneity, chronicity, relapse, multi-organ disease and treatment history	Inflated efficacy estimates and poor reproducibility in clinical development	Multiple complementary models; spontaneous/age-related or large-animal models where appropriate; human organoids and ex vivo tissues	Mechanism-informed translational model portfolios combined with patient-derived systems and multi-omics-guided target validation
Mechanistic fragmentation	Carrier and cargo effects are often conflated; M1/M2 readouts oversimplify macrophage states; collagen reduction may be presented as direct antifibrotic action.	Weak rationale for patient selection, dosing, combinations and long-term disease modification	Unloaded-carrier/free-cargo controls; single-cell/spatial profiling; pathway perturbation; myofibroblast, matrix-turnover and organ-function endpoints.	Mechanistic deconvolution and cell-state-resolved validation before claims of immune reprogramming or antifibrotic action.
Batch variability and manufacturing complexity	Complex synthesis, membrane coating or biological-vesicle production can generate variable size, cargo, surface composition and potency	Inconsistent efficacy/safety and difficulty meeting pharmaceutical quality requirements	Critical quality attributes; batch-release specifications; stability; potency assays; process comparability	Scalable modular manufacturing; standardized membrane/EV processing; fewer nonessential components; robust quality-by-design workflows
Incomplete pharmacokinetic and biodistribution characterization	Many studies emphasize efficacy while underreporting circulation, tissue residence, clearance and metabolite fate	Uncertain dose selection, treatment interval and organ-specific safety	Validated quantitative imaging/bioanalysis; PK/PD modeling; clearance pathways; repeated-dose distribution	Mechanism-linked PK/PD optimization and theranostic or traceable platforms where clinically justified
Regulatory and clinical evidence gaps	Complex nanomaterials require product-specific CMC, critical-quality-attribute, stability, potency and comparability packages; biomimetic/EV systems add source and batch variability.	Slow translation, uncertain comparability and difficulty designing first-in-human studies	GMP-compatible process; CQA specifications; potency assays; stability; comparability after scale-up; regulator-aligned nonclinical package.	Design for manufacturability early; simplify nonessential components; align development with FDA/EMA nanomedicine expectations.
Cost and implementation burden	Sophisticated multifunctional or patient-derived platforms may require expensive manufacturing, specialized storage and complex administration	Limited scalability and reduced accessibility even if biologically effective	Cost-of-goods modeling; shelf-life; transport/storage; administration feasibility	Clinically deployable designs with stable formulations, scalable inputs, practical dosing routes and explicit cost-effectiveness targets

ADME, absorption, distribution, metabolism and excretion; CMC, chemistry, manufacturing and controls; EV, extracellular vesicle; GMP, good manufacturing practice; PK/PD, pharmacokinetics/pharmacodynamics; ROS, reactive oxygen species.

### 4.8 An author-proposed, nonsequential evidence framework for SARD nanomedicine

We propose a conceptual assessment framework comprising five interconnected evidence domains, not a validated ranking system or a universally applicable sequence. The domains are: (i) formulation identity, quality, and batch reproducibility; (ii) pharmacokinetics and quantitative exposure in the relevant tissue or biological compartment; (iii) cellular or molecular target engagement, where such targeting is claimed; (iv) mechanistic attribution, including the roles of carrier, cargo, targeting ligand, and release trigger as applicable; and (v) disease-relevant efficacy, longer-term safety, and manufacturability. These domains were selected to address recurring interpretation and translation gaps identified throughout this Review—not because an externally validated model requires progression from Domain 1 to Domain 5. Their relative importance and the order in which evidence is generated will differ among local depots, systemic nanoparticles, active nanomaterials, vesicles, and nucleic-acid carriers. Formulation quality can influence pharmacokinetic interpretation, while efficacy and toxicology data can inform reformulation; the domains are therefore interdependent and may be evaluated iteratively. This framework is offered as an author-derived checklist for transparent evidence appraisal and hypothesis generation, not as a clinical-readiness score, regulatory standard, or prerequisite pathway that every candidate must follow.

### 4.9 Conclusions

Nano-enabled formulations may solve specific pharmacological problems in selected SARD contexts, but superiority over established disease-modifying treatment cannot be assumed. The strongest preclinical evidence currently concerns particular platforms in RA, SLE/LN, and SSc; evidence in SjD and IIM remains less developed and calls for careful separation of demonstrated findings from proposed designs. Future work should align product-specific formulation quality with appropriate measures of exposure, target engagement when claimed, causal attribution, disease-relevant efficacy, and longitudinal safety. The proposed five-domain assessment framework is deliberately nonsequential and should be adapted to platform, cargo, route, and therapeutic objective. Clinical usefulness will ultimately depend on showing a reproducible benefit–risk and manufacturing advantage over a clinically relevant comparator—not on maximal technical complexity or on claims of universal precision.

## Glossary

ADME: absorption, distribution, metabolism and excretion
APC: antigen-presenting cell
CMC: chemistry, manufacturing and controls
CTGF: connective tissue growth factor
DC: dendritic cell
ECM: extracellular matrix
EPR: enhanced permeability and retention
EV: extracellular vesicle
FLS: fibroblast-like synoviocyte
GMP: good manufacturing practice
IFN: interferon
IIM: idiopathic inflammatory myopathy
IL: interleukin
JAK: Janus kinase
LN: lupus nephritis
MMP: matrix metalloproteinase
MSC: mesenchymal stromal cell
MSN: mesoporous silica nanoparticle
NF-κB: nuclear factor kappa B
NLRP3: NOD-like receptor family pyrin domain-containing 3
NP: nanoparticle
PDGF: platelet-derived growth factor
PEG: polyethylene glycol
PK/PD: pharmacokinetics/pharmacodynamics
PLGA: poly(lactic-co-glycolic acid)
RA: rheumatoid arthritis
RBC: red blood cell
ROS: reactive oxygen species
SARDs: systemic autoimmune rheumatic diseases
sEV: small extracellular vesicle
siRNA: small interfering RNA
SjD: Sjögren’s disease
SLE: systemic lupus erythematosus
SSc: systemic sclerosis
STAT: signal transducer and activator of transcription
Tfh: T follicular helper cell
TGF-β: transforming growth factor beta
Th17: T helper 17 cell
TNF: tumor necrosis factor
Treg: regulatory T cell
